# A prospective hospital study of alcohol use disorders, comorbid psychiatric conditions and withdrawal prognosis

**DOI:** 10.1186/s12991-016-0111-5

**Published:** 2016-08-31

**Authors:** Philippe Nubukpo, Murielle Girard, Jean-Marie Sengelen, Sophie Bonnefond, Aurélien Varnoux, Benoît Marin, Dominique Malauzat

**Affiliations:** 1 Pôle Addictologie, Centre Hospitalier Esquirol, 15 rue du Dr Marcland, 87025 Limoges, France; 2 Département de Recherche et Développement (DRD), Centre Hospitalier Esquirol, 87025 Limoges, France; 3 Faculté de Médecine, UMR/INSERM1094, 2 rue du Dr Marcland, 87025 Limoges, France; 4 Pôle de Psychiatrie Infanto Juvénile, Centre Hospitalier Esquirol, 87025 Limoges, France; 5 Pôle de Psychiatrie Adulte, Centre Hospitalier Esquirol, 87025 Limoges, France

**Keywords:** Alcohol, Psychiatry, Comorbidity, Withdrawal, Abstinence, Psychiatric care

## Abstract

**Background:**

The objective of this study was to describe the profile and alcoholic status of a population with alcohol use disorders (AUD) requesting help from a psychiatric hospital to stop drinking, as well as their clinical outcome and care consumption over the 2 years following the request.

**Methods:**

The visits were conducted at baseline (M0) and at 6, 12, 18 and 24 months (M6, M12, M18, M24). Demographic, clinical and psychometric data [Beck Depression Inventory (BDI), AUDIT questionnaire, Global Assessment of Functioning (GAF) scale], and information regarding the use of psychiatric care and therapeutics were collected.

**Results:**

The 330 subjects included were mostly male, aged 45.2 ± 10.2 years with an employment rate of 55.4 %, living alone (69.1 %), with a psychiatric comorbidity (60.9 %), especially depressive, and with few somatic complications. Their global functioning was poor (GAF score 49.14 ± 15.6), and less than 10 % were addicted to another substance. The abstinence rate at 24 months was 41.4 %, but only 23 % (20) abstained continuously between M0 and M24, and 66.7 % (58) intermittently. The likelihood of abstinence at M24 was greater for females aged over 60 years. The BDI score decreased significantly between M0 and M24. In all, 56.2 % of the participants were re-hospitalized after weaning, but were not integrated in long-term medical care.

**Conclusions:**

Abstinence after alcohol withdrawal fluctuated over time indicating the need for long-term support. The treatment of AUD should not target total, continuous abstinence. Prognostic profiles combining socio-demographic, clinical and biological indicators must be established.

## Background

Over the past few centuries, the French society has treated alcoholism as an affliction in a complex context combining an ancestral way of life with divergent economic, social, political and health interests. Alcohol use disorder (AUD) is a major direct and indirect cause of high rates of mortality and morbidity worldwide [1].

The lifetime prevalence of AUD is estimated to be between 7 and 12.5 % in most Western countries [2, 3]. In primary health care (general practice) in France, the prevalence of problematic alcohol use is estimated to be between 5.2 and 7.9 %, but may reach 11 % for risky or dangerous alcohol consumption [4, 5].

Long-term mortality in alcohol-dependent subjects is three to five times higher than the theoretical mortality calculated for non-alcohol-dependent subjects, and in-hospital mortality caused by health problems linked to alcohol abuse is about 20 % in subjects hospitalized for psychiatric disorders or other diseases [5–9]. The social costs of AUD are high in France and throughout Europe, due to accidents, absenteeism, direct and indirect medical costs and negative impact on the family environment and the living conditions [10–12].

Comorbidity with psychiatric problems is associated with a poorer prognosis of both conditions due to diagnostic problems and difficulties with the care pathway [13–15].

According to the World Health Organization (WHO), comorbidity is defined in psychiatry as “the occurrence, in a given person, of both a problem due to the consumption of a psychoactive substance and another psychiatric problem”. Comorbidity, or dual diagnosis, is a major public health issue for several reasons. The addition of an addictive comorbidity to a psychiatric problem increases the impact of the disease on the patient and their family [16], worsens violent behavior and breakups due to mental problem [17] and increases the financial cost of the disease [16, 18]. The health benefits of weaning patients off alcohol are recognized, but the prognosis after withdrawal of alcohol-dependent subjects with comorbid psychiatric conditions is less well understood. In particular, we know little about the abstinence profile during the months following the cessation of alcohol consumption, or the links between fluctuations in alcohol consumption during this period and clinical data and biological factors. To improve our understanding of AUD in psychiatry, we carried out a cohort study, in a French psychiatric hospital, on 330 alcohol-dependent subjects requesting assistance to stop drinking.

The aim was to establish a profile of the people concerned (those with a high probability of a dual diagnosis of AUD and a comorbid psychiatric condition) and to describe the changes in their alcohol use status over the 2 years following their withdrawal according to clinical and biological criteria, as well as their use of health-care services.

## Methods

This study was carried out at Esquirol Hospital in Limoges, France, a regional public psychiatric hospital with 985 beds and the capacity to accommodate partially or fully hospitalized patients suffering from psychiatric problems and/or addiction. The frequency of AUD in the region and the consultation rates in centers specializing in alcohol-related problems (2.8 inhabitants aged between 20 and 70 years per thousand) were similar to those for the rest of France [19].

### Study population

We included 330 people aged 18 years or over, presenting with AUD with dependence and requesting treatment to stop drinking during the first week of hospitalization in the hospital care units between September 2006 and September 2008. These individuals were included regardless of their mode of alcohol consumption and their psychiatric and/or somatic comorbid conditions. We did not include individuals with no fixed abode, presenting a progressive somatic condition with an estimated life expectancy shorter than 24 months, displaying noticeable alterations of cognitive function preventing the evaluations (illiteracy, dementia or moderate to severe intellectual deficiency), as evaluated by the doctor in the care unit of hospitalization.

### Ethics, consent and permissions

This study was carried out in accordance with French regulations governing biomedical research and was approved by the Research Board of the French Ministry of Health, the Limousin institutional review board [Comité de Protection des Personnes qui se prêtent à une Recherche Biomédicale (CPPRB)] and the French data protection agency (CNIL).

Each participant gave an informed written consent.

### Study design

We avoided interventions that would affect the participants’ outcome: 6-monthly follow-up visits with no major constraints for the participants were scheduled and integrated as closely as possible in the essential care procedures already established for this type of disease.

The investigators were not given any instruction about the type of therapeutics to use during the survey (either pharmacological or non-pharmacological), because of the semi-naturalistic nature of the study. So, the participants received non-pharmaceutical support of their clinician, which consisted of a psychological/psychosocial (BRENDA type) approach. Neither cognitive behavioral therapy nor any other structured intervention was provided. The participants were thus re-evaluated every 6 months, over a period of 2 years (M0, M6, M12, M18, M24), when hospitalized or as outpatients. The various visits were carried out during a medical consultation within the framework of the standard alcohol withdrawal follow-up program. If no medical visit was scheduled for one of the time points, a research nurse contacted the participant by telephone or e-mail to schedule an appointment for a clinical evaluation and to collect the biological samples required for the study. The reasons for follow-up visits not taking place were classified as “lost to follow-up”, “refusal” or “death”. Participants were considered “lost to follow-up” if they could not be contacted, if they did not respond when contacted, or if they were unable to attend (e.g., unable to travel, moved home, illness). Additional efforts were made to contact participants for the final follow-up visit at 24 months (letters, telephone calls, contact made with relatives or family doctor).

### Clinical, psychometric and biological measurements

At inclusion, a clinical evaluation was carried out by the doctor of the unit in which the patient was hospitalized (the consultant doctor). The principal diagnosis and comorbid conditions were identified according to the DSM-IV-TR criteria by the same consultant doctor, together with socio-demographic data (lifestyle, marital status, professional activity, source of income, protective measures), history of psychiatric care (number of years, number of hospitalizations), psychometric data [Alcohol Use Disorders Identification Test (AUDIT), Beck Depression Inventory (BDI), Global Assessment of Functioning (GAF) scale] and current treatment. A blood sample was taken as part of routine care by the unit staff. The complete biological evaluation included measurements of mean corpuscular volume (MCV) and gamma-glutamyl transferase (γGT) activity at all follow-up visits, as well as determination of liver transaminase serum glutamate oxaloacetate transaminase (SGOT) and serum glutamic pyruvic transaminase (SGPT) activity at M0 and M24.

The data collected at the M6, M12, M18 and M24 follow-up visits were, with the exception of the psychiatric care history already recorded, almost identical to those collected at inclusion. The following information during the follow-up visits was collected: alcohol consumption status, GAF score and comorbidities if the patient saw the doctor in the frame of their usual medical care pathway. AUDIT score (at M12 and M24), duration of care received (number of days of partial or full hospitalization), outpatient follow-up (e.g., outpatient care, home visits by nurses), rate of rehospitalization after alcohol withdrawal between M0 and M24, time between therapeutic alcohol withdrawal and first care intervention were non-medical data collected by a research nurse or care staff nurses.

The AUDIT questionnaire [20] addresses current alcohol-related problems and explores behavior over the previous 12 months. Based on the total score, it is possible to classify alcohol consumers according to their type of consumption: risky consumption or use (score strictly below 7 in men, or 8 in women), misuse or harmful use (score between 7 and 12 in men, or between 8 and 11 in women) or alcohol dependence (score strictly greater than 12 in men, or 11 in women).

The 13-item Beck Depression Inventory, or BDI, was used to evaluate depressiveness [21–23]. It yields a score from 0 to 39, which is interpreted as follows: 0–3: no depression; 4–7: mild depression; 8–15: medium to moderate depression; 16 and over: severe depression.

Using the Global Assessment of Functioning (GAF) scale (DSM-IV-TR axis V), clinicians can evaluate the psychological, social and professional functioning of the patient on a hypothetical continuum from full mental health (100 or optimal functioning) to disease (0 or completely non-functional). The evaluator should not take into account changes in functioning due to limiting physical or environmental factors.

Abstinence was determined at M6, M12, M18 and M24 using a combination of clinical and biological criteria: declaration of absence of alcohol consumption by the participant (interview with a doctor or a study nurse) and concordance between a mean corpuscular volume (MCV) below 96 µm^3^ and serum γGT activity at least half that at the time of alcohol withdrawal or below the reference value (<52 IU/ml for men or <32 IU/ml for women, according to analytical laboratory standards). Subjects not satisfying these criteria were considered non-abstinent. Relapse at one of the follow-up visits was determined by objective detection by the investigator, using clinical or biological means of “non-abstinence” after a period of abstinence. Abstinent subjects were those for whom abstinence was clearly established at each of the follow-up visits over the 24-month period. Intermittently abstinent subjects at M24 were defined as subjects for whom alcohol consumption was detected during at least one of the follow-up visits, but with abstinence at M24.

To determine the care pathway, the number of days of partial or full hospitalization was extracted from the computerized patient data records, which included medical and paramedical interventions, the number of days in hospital for each individual seen at the hospital and, depending on the information provided by the participants, admissions to other psychiatric or non-psychiatric facilities. The cumulative duration of these sequences was calculated in days for the 24-month follow-up period. Outpatient interventions were also recorded.

### Statistical analysis

The results are presented as means ± standard deviations for continuous quantitative variables (age, BDI score, AUDIT score, GAF score, γGT levels, MCV, SGOT and SGPT levels, number of care interventions, duration of care sequences, etc.) and as percentages and absolute numbers for qualitative variables (sex, abstinence status, lifestyle, social welfare benefits, type of diagnosis, presence of comorbid conditions, socio-demographic data). The nonparametric Kruskall–Wallis test (or the Mann–Whitney test for pairwise comparisons) was used to compare the distributions of quantitative variables between groups, as these variables were not normally distributed. Differences in data between M0 and the other follow-up visits were compared using nonparametric paired Wilcoxon tests.

Student’s *t* test was used to compare quantitative variables (mean scores on the Beck and AUDIT scales) between subgroups, because these variables followed a normal distribution. Pearson’s *χ*^2^ test was used to compare qualitative variables between subgroups, or Fisher’s exact test if the number of values in each cell was too small. We used Systat version 11 for Windows and *p* < 0.05 was considered to be significant.

We carried out a logistic regression analysis to identify the variables associated with abstinence, with the aim of determining the reasons for alcohol abuse. We carried out an initial series of univariate logistic regression analyses. The variables considered in the univariate analysis were diagnostic category at M0, type of care (outpatient, hospitalization), age group (<30, 30–60, >60 years), sex (male/female), living alone (yes, no) and on social welfare benefits (yes/no). The variables with a *p* value less than 0.2 in the univariate analysis were included in a multivariate logistic model. Qualitative variables were integrated in the model without modification. Prior checks were carried out for the quantitative variables: if the odds ratio was demonstrated to be log-linear, then these variables were included in the model without modification; otherwise, they were categorized by quartile. The initial multivariate model was simplified by a stepwise backward elimination method, such that the final model included only variables significantly associated with the variable to be explained. We checked that no confounding occurred during the stepwise elimination procedure. Following the univariate analysis on the study population of 246 people, the five variables retained for the multivariate analysis were care category, age group, sex, living alone (yes, no) and on social welfare benefits category. This part of the analysis was carried out using SAS software (SAS V9.3, SAS Institute, Cary, NC).

## Results

### General description of the cohort at inclusion

Socio-demographic, clinical and biological data for the participants at inclusion are summarized in Table 1.

**Table 1:** Characteristics of the population at inclusion

*N* = 330, unless otherwise indicated	Mean ± SD (*n*) or % (*n*)
Age (years)	45.2 ± 10.2
Sex-ratio (male/female)	3.6 (258/72)
Single/divorced/widowed (324)	69.1 % (224)
Lifestyle (324)	
Couple	31.5 % (102)
Living alone	39.1 % (127)
Other (family, friends…)	29.4 % (97)
With a professional activity	55.4 % (174)
Resources (327)	
Own or from family	67.9 (222)
Social	32.1 (105)
Smokers	80.2 % (186)
With psychiatric co-morbidity	60.9 % (200)
Type of psychiatric comorbidity	
None	39.1 % (129)
Dementia	1.5 % (5)
Psychosis	4.6 % (15)
Mood and affective disorder	30.6 % (101)
Anxiety disorder	5.5 % (18)
Personality disorder	26.1 % (87)
With somatic comorbidity	7.6 % (25)
Global Assessment of Functioning	49.14 ± 15.60
AUDIT (312)	26.9 ± 7.7
Risk use (score ≤7)	2.2 (7)
Harmful use (7< score <13)	2.2 (7)
Dependence (score ≥13)	95.5 (298)
BDI score (308)	13.5 ± 7.1
BDI categories	
Absent	5.8 (18)
Slight	16.2 (50)
Moderate	41.6 (128)
Severe	36.4 (112)
MCV (µm^3^)	97.9 ± 6.2
% >normal values (*n*)	55.9 (181)
SGOT (UI/L)	47.4 ± 53.5
% >normal values (*n*)	39.3 (129)
SGPT (UI/L)	42.5 ± 41.5
% >normal values (*n*)	41.5 (136)
GGT (UI/L) (mean ± SD)	222.7 ± 408.2
% >normal values (*n*)	61.6 (202)
Current psychotropic treatment	
Neuroleptics	14.2 % (47)
Atypical antipsychotics	14.5 % (48)
Thymoregulators	7.8 % (26)
Antidepressants	52.7 % (174)
Anxiolytics	90 (297)
Benzodiazepines	88.4 % (292)
Related to benzodiazepines	23.0 % (76)

*AUDIT* alcohol use disorder identification test, *BDI* Beck Depression Inventory, *GGT* gamma-glutamyl transferase, *MCV* mean corpuscular volume, *SGOT* serum glutamate oxaloacetate tranasminase, *SGPT* serum glutamic pyruvic transaminase

The study population consisted largely of young men, mostly living alone (69.1 %). About one-third of the participants declared living with someone else as a couple. Just over half the participants were employed, and most of their income came from private sources rather than from benefits.

In terms of addictive comorbid conditions, smoking was highly prevalent. In contrast, few subjects were dependent on another psychoactive substance: 2.5 % were dependent on cannabis, 0.8 % on opiates and 0.4 % on cocaine.

In terms of psychiatric comorbid conditions, depression was the most frequent comorbid condition (*p* < 0.001), followed by psychotic and anxious disorders.

According to the AUDIT scores, seven had a score of 7 or below. Clinical examinations confirmed alcohol dependence in these 14 cases. Mean daily consumption (TAC) was high, 34.6 % (108 people) of the participants declared consuming at least 10 units per day and 51.6 % (161 people) 5–8 units per day. Similarly, the number of heavy drinking days corresponded to “almost every day” for 51 % of the participants (159 people). The mean BDI score indicated moderate to severe depressiveness for this population, with a total proportion of 78 % of those included presenting a high score for depression.

Only a few somatic comorbid conditions were reported: ankylosing spondylitis (3 cases), esophagus, stomach and duodenum diseases (4), respiratory diseases (5), liver disease (1), viral infections (3), heart disease (3), metabolic disorders (2), epilepsy (2), breast cancer (1) and other physically disabling conditions (4 cases).

The values for biological markers of liver function and alcohol dependence (γGT and MCV) were both above the normal range (reference values) for 42 % of the participants (136 people). The values for these two markers were in the normal range for 24.7 % of the participants (80 people).

Regarding drug therapy, the most frequently prescribed drug at inclusion was benzodiazepine, followed by antidepressants, which were taken by more than half the participants.

### Changes over the 24 months following the request for help to stop drinking and abstinence

Data were collected for about half the patients at the M6, M12 and M18 visits (Table 2).

**Table 2:** Quantitative and qualitative variables collected at 6 (M6), 12 (M12), 18 (M18) and 24 (M24) months after alcohol withdrawal

	M6		M12		M18		M24	
	Mean ± SD or %	*N*	Mean ± SD or %	*N*	Mean ± SD or %	*N*	Mean ± SD or %	*N*
With any data	51.8	171	38.6	146	42.7	141	51.8	249
With data to assess abstinence	27.6	91	33.9	112	33.9	112	75.5	171
Abstinent	57.1	52	40.2	45	46.4	52	41.4	103
Reasons for the absence of follow-up								
No data—lost	47.8	76	62.4	118	38.8	128	13.3	13
Impossible to come	24.8	82	34.4	65	15.2	50	19.4	64
Death	0.03	1	3.2	6	7.3	14	21.4	21
With a psychiatric comorbidity	56.7	97	63.7	93	42.5	77	48.2	120
Type of psychiatric comorbidity					37.7			
None	29.9	29	42.0	39	5.19	29	46.7	50
Dementia	0	0	0	0	15.5	4	0	0
Psychosis	9.3	9	18.2	17	20.7	12	14.9	16
Mood disorder	37.1	36	20.4	19	3.9	16	13.1	14
Anxiety disorder	9.3	9	3.2	3	24.7	3	1.9	2
Personality disorder	21	18	20.4	19		19	15.9	17
With somatic comorbidity	11.3	11	6.5	6	5.2	4	5.6	6
Global Assessment of Functioning	59.9 ± 19.7	70	56.7 ± 20.1	66	59.7 ± 20.6	63	58.2 ± 19.3	88
AUDIT score	–		14.5 ± 12.2	130	–		14.0 ± 12.1	162
Risk use (score ≤7)	–		36.2	45	–		40.1	65
Harmful use (7 < score <13)	–		13.1	17	–		6.2	10
Dependence (score ≥13)	–		50.8	66	–		50.6	82
BDI	8.3 ± 7.3	163	7.8 ± 7.5	133	7.2 ± 7.1	130	7.7 ± 7.3	168
Absent	29.5	48	39.1	52	36.9	48	40.2	68
Slight	25.8	42	15.8	21	24.6	32	20.1	34
Moderate	28.8	47	30.1	40	25.4	33	21.3	36
Severe	15.9	26	15.0	20	13.1	17	18.3	31
MCV (UI/L)	94.9 ± 6.0	175	95.4 ± 5.9	140	94.4 ± 5.9	141	94.3 ± 6.2	169
% >normal values (*n*)	34.9	61	40	56	30.5	43	34.3	58
TGO (UI/L)	–		–		–		39.0 ± 55.3	
% >normal values (*n*)							24.6	41
TGP (UI/L)	–		–		–		38.8 ± 61.9	
% >normal values (*n*)							29.9	50
GGT (UI/L)	94.9 ± 6.0	175	108.0 ± 269.3	143	112.9 ± 290.8	140	156.0 ± 379.9	168
% >normal values (*n*)	43.4	76	40.6	58	41.4	58	18.8	82
Current psychotropic treatment								
Neuroleptics	15.3	27	13.3	20	18.0	27	15.0	36
Atypical antipsychotics	25.0	44	24.6	37	24.0	36	23.0	55
Thymoregulators	13.6	24	12.0	18	12.0	18	11.3	27
Antidepressants	63.0)	111	65.3	98	62.6	94	51.8	124
Anxiolytics	67.0	118	65.3	98	61.3	92	57.3	137
Benzodiazepines	56.2	99	56.0	84	11.5	38	48.9	116
Related to benzodiazepines	22.1	39	17.3	26	25.1	83	18.1	43

*AUDIT* Alcohol Use Disorder Identification Test, *BDI* Beck Depression Inventory, *GGT* gamma-glutamyl transferase, *MCV* mean corpuscular volume, *SGOT* serum glutamate oxaloacetate transaminase, *SGPT* serum glutamic pyruvic transaminase

However, data were obtained for a larger proportion of the patients at M24, presumably due to the greater effort made to contact participants and, if the participants themselves could not be contacted, their families. The availability of biological data, and thus knowledge about abstinence, was more limited as it was restricted to patients we were able to trace.

The differences between the population at inclusion and the population at M24 correspond to the difference in the proportion of subjects living alone (35.2 % at M0 and 37.7 % at M24, *p* < 0.001), being single/widowed or divorced (34.5 % at M0 versus 31.3 % at M24; *p* < 0.001), having a professional activity (57.6 % at M0 and 41.2 % at M24, *p* < 0.001) and being on benefits (71.4 % at M0 versus 60.1 % at M24, *p* < 0.001). Neither age (45.2 ± 10.2 years versus 45.6 ± 10.2 years, *p* = 0.12) nor sex (78.7 % men at M24 versus 78.2 % at M0, *p* = 0.681) differed between M0 and M24.

A large number of subjects (21) died during the 24 months of the study.

Overall, the abstinent individuals did not follow a linear trajectory between M0 and M24. Data for all the follow-up visits were available for 87 people, 58 of whom (66.7 %) displayed intermittent abstinence. Only 23 % (20 participants) were entirely abstinent during the entire period from M0 to M24. Clinical data, but without biological data, were obtained for 281 participants; 24.6 % (69 participants) declared a total absence of alcohol consumption over the entire 24-month follow-up, whereas 61.5 % reported intermittent alcohol consumption.

The number of subjects remaining abstinent varied according to the criteria: clinical or biological. An analysis of the coherence of the two definitions of abstinence yielded a kappa value of 0.2307, with a 95 % confidence interval of 0.08–0.38. These values indicate poor concordance between the two measures.

We considered all records of diagnoses of comorbid conditions at any time point during the care of the subject within the 24 months following alcohol withdrawal. Mood disorders were the most frequently reported. The type and nature of the psychiatric co-morbidities reported at M24 differed from those at M0 (*p* < 0.001) with significantly less subjects with mood disorders or anxiety disorders, but an equivalent proportion of psychotic disorders, suggesting that this category benefits from better insertion in the care system.

Few somatic comorbid conditions were reported during the follow-up. At the various follow-up visits, we recorded all diagnoses for diverse conditions requiring treatment and causing some disability (e.g., lower back pain) (*n* = 5), episodic conditions (2), viral infections including hepatitis (9), lung and ear diseases (2), metabolic problems (obesity) (1) and cirrhosis (1). The proportion of somatic conditions reported between M0 and M24 did not differ (*p* = 0.516).

The mean scores for the psychometric scales indicated an improvement in the general state of the participants from M6 onward. At each follow-up visit, we observed an improvement in GAF score (*p* < 0.01), a significant decrease in depression score (*p* < 0.001) and a significant decrease in alcohol dependence (AUDIT score; *p* < 0.001) with respect to the values obtained at M0. The values obtained at the various follow-up visits (M6, M12, M18 and M24) did not differ for the population as a whole. In addition, the mean values of the biological markers used (γGT and MCV) returned to normal during the follow-up, whereas they were outside this range at M0.

Before requesting assistance to stop drinking, the participants had been hospitalized more than four times on average (Table 3). Only 39 % (129 participants) were admitted to a psychiatric unit for the first time when they requested assistance to stop drinking. Similarly, the mean duration of psychiatric care before the request for assistance was relatively short, although 19 % of the patients had been receiving care for over 10 years.

**Table 3:** Psychiatric care before inclusion to the study (before alcohol withdrawal) and during the 24 months after alcohol withdrawal (between M0 and M24) (*n* = 330) [(mean ± SD or % (*n*)]

Before alcohol withdrawal	
Number of psychiatric hospitalizations	4.4 ± 8.3
Duration of psychiatric care (years)	4.5 ± 7.1
Between M0 and M24	
Cumulative number of days of complete hospitalization	79.8 ± 130
Cumulative number of days of psychiatric home care	52.4 ± 71.9
Rehospitalization	56.2 % (164)
Number of days between withdrawal and first psychiatric care	146.4 ± 162.8
Home visits	1.5 ± 8.4

Almost half the participants were readmitted in the 24 months following withdrawal, whereas a quarter only had outpatient follow-up (23.6 %). Patients returned to the care system relatively rapidly, with rehospitalization or the first care intervention within the first 6 months after weaning off alcohol. A total absence of care in the 24 months after weaning off alcohol was observed for only 7 % of the participants (Table 3).

We further studied the psychiatric comorbid conditions and characteristics according to care data. Patients with psychiatric comorbid conditions attended more follow-up visits for this study than patients without such comorbid conditions. For example, 51.4 % (90 participants) of subjects with comorbid conditions attended at least three follow-up visits, in comparison with only 32.3 % (50 participants) of those without comorbid conditions (*p* = 0.011). The first care intervention was between M0 and M6 for 70.9 % (124 participants) of subjects with comorbid conditions, but only 62.6 % without comorbid conditions (*p* < 0.001). A total absence of treatment was observed for only 13.1 % (23 participants) of subjects with comorbid conditions and 25.8 % (40 participants) with no psychiatric comorbid condition.

We looked for an association between socio-demographic variables (age, sex, source of income, living alone) or care received during the 24-month follow-up (hospitalizations, outpatient care) with abstinence status at M24.

Following the univariate analysis of the study population, the five variables retained for the multivariate logistic regression were care category (*p* = 0.199), age (*p* = 0.015), sex (*p* = 0.033), living alone (*p* = 0.146) and private income (*p* = 0.118).

In the multivariate logistic model, age and sex were significantly associated with abstinence at 24 months, as older age was associated with a higher likelihood of abstinence at 24 months. Participants aged over 60 years were therefore six times more likely to be abstinent at the end of the 24-month follow-up than participants under the age of 30 years. Women were twice as likely as men to be abstinent after the 24-month follow-up (Table 4). At the end of the manual stepwise descending procedure for logistic regression, only age was still included in the adjusted model.

**Table 4:** Odds ratios (OR) unadjusted and adjusted (logistic regression) for the association of age and gender to abstinence at 24 months after alcohol withdrawal

Effect	Unadjusted			Adjusted		
	Odds ratio	95 % confidence interval		Odds ratio	95 % confidence interval	
Ambulatory care	0.93	0.36	2.42			
Hospitalization	0.58	0.25	1.37			
Age <60	2.10	0.66	6.66	2.10	0.66	6.66
Age >60	7.58	1.74	33.09	7.58	1.74	33.09
Gender female/male	1.98	1.06	3.70			
Living alone	0.67	0.39	1.15			
Own resources	1.59	0.89	2.84			

## Discussion

### Profile on inclusion

The study population was mostly male (78 % men) and was therefore comparable to the populations in epidemiological studies carried out in the USA: the National Comorbidity Survey [24, 25] and the National Epidemiologic Survey on Alcohol and Related Conditions (NESARC cohort study) [26]. Marital status (69 % were single, divorced or widowed) differed significantly from that of the general population in the region (32.5 % single, 7 % divorced, 10.5 % widowed, corresponding to 50 %) [27]. Insertion in the workplace was better than expected for individuals requesting care in a psychiatric unit, because psychiatric problems are known to be a factor favoring employment loss and a low likelihood of returning to work [28]. However, the cohort employment rate (55.4 %) was lower than that of the general population in Limousin (70 % of 15- to 49-year-olds and 51 % of 50- to 64-year-olds work) [29] or in France (65.1 % of the French population was in paid work in March 2008, and the current proportion is 64.1 %). The GAF score [30], which measures overall functioning, improved over the 24-month follow-up.

The AUDIT score has a considerably valuable number of features for the routine diagnosis of risky or dangerous alcohol consumption. However, it is less relevant for follow-up as it was designed as a screening test [20]. Nevertheless, at least three of the items, those concerning the frequency of alcohol consumption, total alcohol consumption and the number of heavy drinking days (at least six units) decreased between M0 and M24 in our study, for subjects with high consumption levels at M0. This is particularly relevant as decreases of only a few units for “heavy drinkers” have been shown to have an exponential effect on damage reduction [31].

The mean BDI score for the cohort was high for a population not included on the basis of depressive illness. However, this scale only evaluates the intensity of symptoms associated with depression and has no diagnostic value.

Depressive mood is very frequent immediately after alcohol withdrawal, potentially due, at least in part, to the high frequency of depressive symptoms in this population. The decrease in BDI depression scores for all patients after 6 months of follow-up reflects the positive consequences of a decrease in alcohol consumption on depressive symptoms [5, 32]. More than half the participants presented a dual diagnosis of AUD and another psychiatric problem. The frequency of psychiatric problems among alcohol-dependent patients in the general population has already been shown to be high [33–35] and is even higher in hospitalized populations [36]. This may reflect the greater demand for assistance to stop drinking by patients with comorbid conditions, otherwise known as Berkson’s bias [37, 38]. This effect was amplified in our study by the recruitment of participants from a psychiatric hospital environment. In our study population, depression was the most frequent comorbid psychiatric condition, ahead of psychotic, anxious and bipolar disorders, both at inclusion and after 24 months. Some of the patients initially diagnosed as having depression in this cohort may actually have had bipolar depression, and this might result in a need to readjust the diagnoses during the follow-up period. Previous studies have highlighted the difficulties associated with these diagnoses in alcohol-dependent patients [39, 40].

Most studies have shown that depressive symptoms are frequent in people with AUD, but that characterized depressive episodes are rare. These transient symptoms of depression not fulfilling the criteria for diagnosis of a major depressive episode disappear after 1–2 weeks of abstinence [41, 42] and should not lead to treatment with an antidepressant unless they persist for much longer [15].

The high death rate of 6.4 % (21/330) among the patients seeking a cure for alcohol dependence during the survey can be emphasized. The absolute risk of dying from an adverse alcohol-related condition increases linearly with the amount of alcohol consumed over a lifetime [43]. The annual absolute risk of dying from an alcohol-related disease for people aged over 15 years across the population in the World Health Organization (WHO) European Region is around 0.1 for a daily consumption of over 70 g. In 2012, 5.9 % of all global deaths were attributable to alcohol, with the highest proportion in the WHO European Region [43].

Finally, we observed an increase in the prevalence of psychiatric problems during the course of the study, potentially corresponding to an initial underestimation of comorbid psychiatric conditions and the occurrence of new clinical episodes of psychiatric conditions during the 2-year follow-up period [1, 24, 25]. The clinical subpopulation with a dual diagnosis of AUD and a psychiatric problem should be studied in greater detail, because such dual diagnoses have been shown to be associated with a poorer prognosis for both conditions [14, 33, 34, 44].

### Abstinence and changes during the 24 months following the request for assistance to stop drinking

Overall, 41.4 % of the subjects were still abstinent at 24 months. However, focusing solely on abstinence at this last time point (M24) does not provide an entirely satisfactory evaluation of the success of the intervention. Our results also raise questions regarding the criteria used to evaluate abstinence. The use of a combination of clinical and biological criteria for abstinence makes it possible to optimize the detection of “false abstinent” individuals. However, in practice, these criteria may be too strict, potentially masking real differences. The use of abstinence as the only criterion for evaluating care in the treatment of patients with AUD is now seen as somewhat heavy handed. Decreasing consumption also seems to be a beneficial objective, with the aim of decreasing morbidity and mortality [45–47]. Finally, old age (over the age of 60 years) and being female were the only criteria significantly linked to successful weaning off alcohol after 24 months in our study. However, this finding should be interpreted with caution given the small proportions of women and subjects over the age of 60 years in our study population. Nevertheless, these individuals may constitute subgroups of the population with more difficult access to care for AUD and, thus, with probably a much greater motivation to succeed.

The request for help to stop drinking is part of a long, very personal process. It is invariably preceded by a long progressive phase that is not sufficient in itself to create the conditions required for sustained abstinence. This process involves alternating periods of alcohol consumption and partial or complete abstinence, sometimes linked to identifiable factors, but above all to conflicts and internal thought processes.

Sustaining medium- and long-term abstinence seems to be directly linked to the attention devoted to the addiction problem, the psychiatric conditions with which it is comorbid and the quantity and pertinence of care delivered [33, 48]. Patients with comorbid psychiatric conditions were more compliant in this study. This may have been due to their stronger demand for help or prior integration in a follow-up system, thereby facilitating follow-up for this study. This was the case, for example, for patients with schizophrenia and AUD. For the patients, the follow-up was part of the care process. It has also been reported that patients with a dual diagnosis make greater use of care facilities [49], including emergency services [16]. In the context of care rationalization, changes in the use of care facilities over time should be considered in more detail. In contrast, few of the interventions by psychologists and social workers took place in the patients’ homes, presumably reflecting the largely institutional and medical management of AUD in France. The results provided here give an evidence of the patients—institution interaction, which does not appear to be so intensive and tightened.

Antidepressant use was high (52.7 %). In France, recommendations are to wait for 2–4 weeks after weaning off alcohol before introducing antidepressant treatment, unless the symptoms are acute and intense with a risk of suicidal behavior. Furthermore, benzodiazepines, which are authorized for use as anxiolytics for short periods of no longer than 12 weeks, should not be prescribed for longer than the standard duration of the weaning period, which is of the order of about 10 days at most [50]. It can be highlighted that the investigators were not given any instructions regarding the type of therapeutics to use during the survey (either pharmacological or non-pharmacological) because of the semi-naturalistic nature of the study,

### Bias

The results obtained in this study were collected for a specific population: chronic alcohol consumers who had contacted the hospital’s psychiatric department. Overall, 20.30 % of the patients were lost to follow-up at M24, but not all the patients attended all the follow-up visits. This limited the sample size, with deleterious consequences for the statistical analysis. Indeed, this study was semi-naturalistic in design, with data collection based largely on the integration of participants in a care pathway that varied from patient to patient. Nevertheless, we consider this one of the strengths of the cohort, as it includes strict evaluations linked to the study of a fate more compatible with a patient-focused care pathway for subjects requesting help from the psychiatric care system to stop drinking.

## Conclusion

In this observational cohort study of 330 patients requesting assistance from the psychiatric care system to stop drinking, we found that only 25 % of the participants remained abstinent throughout the entire 24-month follow-up, with 41.4 % of the participants considered abstinent after 2 years; 66.7 % of the participants displayed intermittent abstinence over the 2-year follow-up period. Some sub-groups may be defined with regard to their chance of abstinence and further characterization may be helpful in the routine practice. However, patients with a comorbid psychiatric condition underwent a larger number of follow-up visits for the study, together with a larger number of treatment sequences and health-care interventions. The lack of agreement between clinical and biological data for abstinence and the other results obtained suggest that studies of combined markers or prognosis, biopsychosocial outcome and fate after weaning are required. These findings also confirm the importance of not focusing solely on short-term management and abstinence.

## Abbreviations

AUD: alcohol use disorders
AUDIT: Alcohol Use Disorders Identification Test
BDI: Beck Depression Inventory scale
DSM-IV-TR: Diagnostic And Statistical Manual of Mental Disorders IV Revised
γGT: gamma-glutamyl transferase
GAF: Global Assessment of Functioning
HDD: heavy drinking days
MCV: mean corpuscular volume
SGPT: serum glutamic pyruvic transaminase
SGOT: glutamate oxaloacetate transaminase
TAC: total alcohol consumption
WHO: World Health Organization
